# Expression of CC chemokine receptor 9 predicts poor prognosis in patients with lung adenocarcinoma

**DOI:** 10.1186/s13000-015-0341-x

**Published:** 2015-07-11

**Authors:** Yonglong Zhong, Lingyu Jiang, Hui Lin, Baijun Li, Jiao Lan, Shengjing Liang, Bin Shen, Zhenniu Lei, Weiping Zheng

**Affiliations:** Department of Thoracic Cardiovascular Surgery, the People’s Hospital of Guangxi Zhuang Autonomous Region, No. 6 Taoyuan Road, Nanning, 530021 P.R. China; Intensive Care Unit, the People’s Hospital of Guangxi Zhuang Autonomous Region, Nanning, 530021 P.R. China; Research Center of Medical Sciences, the People’s Hospital of Guangxi Zhuang Autonomous Region, Nanning, 530021 P.R. China; Provincial Clinical Medical College of Fujian Medical University, Fuzhou, 350000 P.R. China

**Keywords:** Chemokine receptor 9, Aggressive behavior, Lymph node metastasis, Prognosis, Lung adenocarcinoma, Immunohistochemistry

## Abstract

**Background:**

The CC chemokine receptor 9 (CCR9) plays an important role in tumorigenesis and metastasis in various cancers. Our previous studies have shown the aberrant expression of CCR9 in non-small cell lung cancer (NSCLC) cell lines, revealing that the CCR9-CCL25 axis modulates cell migration and invasion, and supports cancer cell survival by inhibiting apoptosis *in vitro* and *in vivo*. In the present study, we aimed to evaluate the expression and possible prognostic role of CCR9 in lung adenocarcinoma.

**Methods:**

Immunohistochemical analysis of CCR9 expression was performed on 144 lung adenocarcinoma tissues and 30 adjacent normal lung parenchymal tissues. We assessed the correlation of CCR9 expression with clinicopathological characteristics and the prognosis of lung adenocarcinoma.

**Results:**

The expression of CCR9 was increased in lung adenocarcinoma tissue compared with normal lung tissue. Moreover, such an expression was positively correlated with tumor size (p = 0.032), lymph node metastasis (p = 0.002) and advanced TNM stage (p = 0.012). In addition, the patients with negative CCR9 expression exhibited a higher overall survival (OS) compared with those with positive CCR9 expression. Multivariate analysis showed that the CCR9 expression was an independent prognostic factor for the OS of patients with lung adenocarcinoma.

**Conclusions:**

We, for the first time, reported that CCR9 could be beneficial in predicting lymph node metastasis, and it might act as a novel prognostic biomarker for lung adenocarcinoma.

## Background

Lung cancer is the leading cause of malignancy-related deaths for both men and women worldwide [1]. Non-small cell lung cancer (NSCLC) is the predominant component of lung cancer cases. As the most frequent subtype, lung adenocarcinoma accounts for approximately 40 % of all NSCLC cases, and it has a poorer prognosis relative to lung squamous cell cancer. Despite the great progress has been made in the diagnosis and surgical treatment of lung cancer in recent decades, the overall tumor-free 5-year survival rate for human lung adenocarcinoma remains exceedingly low (below 18 %) due to metastatic relapse [2]. Nevertheless, the molecular mechanisms and factors involving in the process of adenocarcinoma pathogenesis and metastasis are not fully elucidated. Therefore, it is necessary to identify novel molecular biomarkers, which may help in providing new therapeutic targets and improving the survival of patients with lung adenocarcinoma.

As members of a superfamily of small inflammatory or homeostatic cytokines, chemokines share a common biological activity in stimulating the migration of different types of cells, including lymphocytes, monocytes, neutrophils, endothelial cells and mesenchymal stem cells [3]. Biologically, in addition to leukocyte recruitment, chemokine receptors and its corresponding ligands are associated with tumor progression, angiogenesis and metastasis [4, 5]. In recent years, chemokine receptors have been recognized as new potential targets or agents in cancer therapy and immunotherapy, suggesting their multifaceted role in the development and progression of cancer [6].

CC chemokine receptor 9 (CCR9) is a G-protein-coupled receptor for the thymus-expressed chemokine (TECK) or CCL25, and the latter one is the only natural ligand of CCR9 [7]. CCR9 is preferentially expressed on subsets of naive T cells and mature dendritic cells, and it was initially identified for its role in immune homeostasis, which is responsible for recruiting immune cells [8]. Notably, many different reports have shown that CCR9 is highly expressed in various cancer cells and tumors, functioning as a critical potential mediator in cancer progression [9]. Furthermore, in our previous study, for the first time, we have shown the potential role of CCR9 in lung cancer, demonstrating that it is significantly over-expressed in tissues and cell lines from NSCLC, and CCR9-CCL25 axis promotes the migration and invasion capability of NSCLC cells *in vitro* [10]. Our subsequent study suggested that CCR9-CCL25 interaction supports lung cancer cell survival and up-regulates the anti-apoptotic signaling mediated by the PI3K/Akt survival pathway [11].

Therefore, we focus on the clinical significance of the CCR9-CCL25 biological axis, which appears to be associated with both good and bad outcomes in cancer prognosis. No studies have examined the expression profiles of CCR9 in lung adenocarcinoma patients with curative resection and its effect on lung adenocarcinoma survival. In the present study, we aimed to investigate the expression of CCR9 in lung adenocarcinoma tissue and its correlations with clinicopathological characteristics. Moreover, we attempted to evaluate the relationship between CCR9 expression and post-operative prognosis.

## Methods

### Patients and tissue samples

Formalin-fixed, paraffin-embedded specimens of 144 lung adenocarcinoma tissue were collected from the People's Hospital of Guangxi Zhuang Autonomous Region (Nanning, P. R. China) between 2008 and 2012. Paired normal lung parenchyma specimens were collected from adjacent tissues (> 5 cm away from tumors) of 30 randomly selected cases. The median age of these 144 patients with lung adenocarcinoma, including 95 males and 49 females, was 58 years old ranging from 32 to 78. All the patients underwent complete pulmonary lobectomy or segment resection and simultaneously received hilar and/or mediastinal lymph node dissections. Pathological staging was performed for all cases according to the revised 7^th^ Edition of the Union for International Cancer Control (UICC). There were 57 cases in stage I, 31 cases in stage II and 56 cases in stage III. Pathological diagnosis was confirmed by two independent pathologists in the Department of Pathology of the People’s Hospital of Guangxi Zhuang Autonomous Region according to 2009 WHO/IASLC. None of the patients received radiotherapy or chemotherapy before surgical resection. A total of 73 patients were treated with routine adjuvant platinum-based chemotherapy after surgery. Our study protocol was approved by the Ethics Committees/Institutional Review Boards of the People’s Hospital of Guangxi Zhuang Autonomous Region, and written informed consent was obtained from every participant.

### Immunohistochemistry

Envision method was employed to detect the immunohistochemical expression of CCR9 on formalin-fixed, paraffin-embedded tissue sections. Briefly, the sections were baked at 65 °C for 60 min, then successively deparaffinized, dehydrated and rinsed with phosphate-buffered saline (PBS). Subsequently, sections were processed in citrate buffer (0.01 M, pH 6.0) and treated with high-pressure antigen retrieval, followed by treatment with 0.3 % hydrogen peroxidase for 10 min to quench the endogenous peroxidase activity. After the sections were washed with PBS, they were incubated with rabbit anti-CCR9 polyclonal antibody (1:100; Abcam, Cambridge, MA, USA) overnight at 4 °C. Then the slides were respectively incubated with polymer helper (PV9000 kits, Zhongshan Bio Corp., Beijing, China) and poly peroxidase-anti-mouse/rabbit IgG antibody (PV9000 kits, Zhongshan Bio Corp., Beijing, China) at room temperature for 30 min. After the incubation, the sections were washed with PBS and developed with diaminobenzidine (DAB) (Zhongshan Bio Corp., Beijing, China) at room temperature for 5 min. Finally, the sections were counterstained with hematoxylin (Sigma, MO, USA), dehydrated and then mounted. For negative controls, the primary antibody was replaced by PBS. The sections with positive CCR9 expression severed as positive controls, and only cytoplasmic staining was considered positive.

### Evaluation of immunohistochemical staining

The immunostained slides were independently assessed by two experienced pathologists blinded to the clinicopathologic information. Briefly, representative areas of each section were selected, and cells were counted in five fields at a 400-fold magnification. Scores for the tumor cells stained positive were defined as follows: 0, ≤ 5 %; 1, > 5 to 25 %; 2, > 25 to 50 %; and 3, > 50 %. Staining intensity was graded according to the following criteria: 0, no staining; 1, weak staining; 2, moderate staining; and 3, strong staining. Based on the semi-quantitative score calculated by multiplying these two values (which ranged from 0–9), the stained sections were defined as either negative expression (0) or positive expression (1–9).

### Follow-up

Follow-up visits were postoperatively scheduled at intervals of 1 month, 6 months, first year, 2 years and annually after surgery, respectively. Follow-up data were mainly obtained through special outpatient reexamination, letters or telephones. Over survival (OS) was defined as the interval between the date of surgery and the date of the death or the date of last visit (if death did not occur). Complete follow-up, ranging from 1 to 86 months, was available for all patients, and the median survival was 32 months.

### Statistical analysis

Associations between CCR9 expression and clinicopathological parameters were assessed using the χ^2^ test as well as the Fisher's exact test. Kaplan-Meier method with the log-rank test was applied for survival analysis. Multivariate analysis of the variable influence on OS was performed using the Cox-proportional hazards model. All statistical analyses were performed using the SPSS 18.0 software package (SPSS Inc., Chicago, IL, USA). All p-values were two-sided, and p < 0.05 was considered as statistically significant.

## Results

### CCR9 is highly expressed in lung adenocarcinoma

**Fig. 1:**
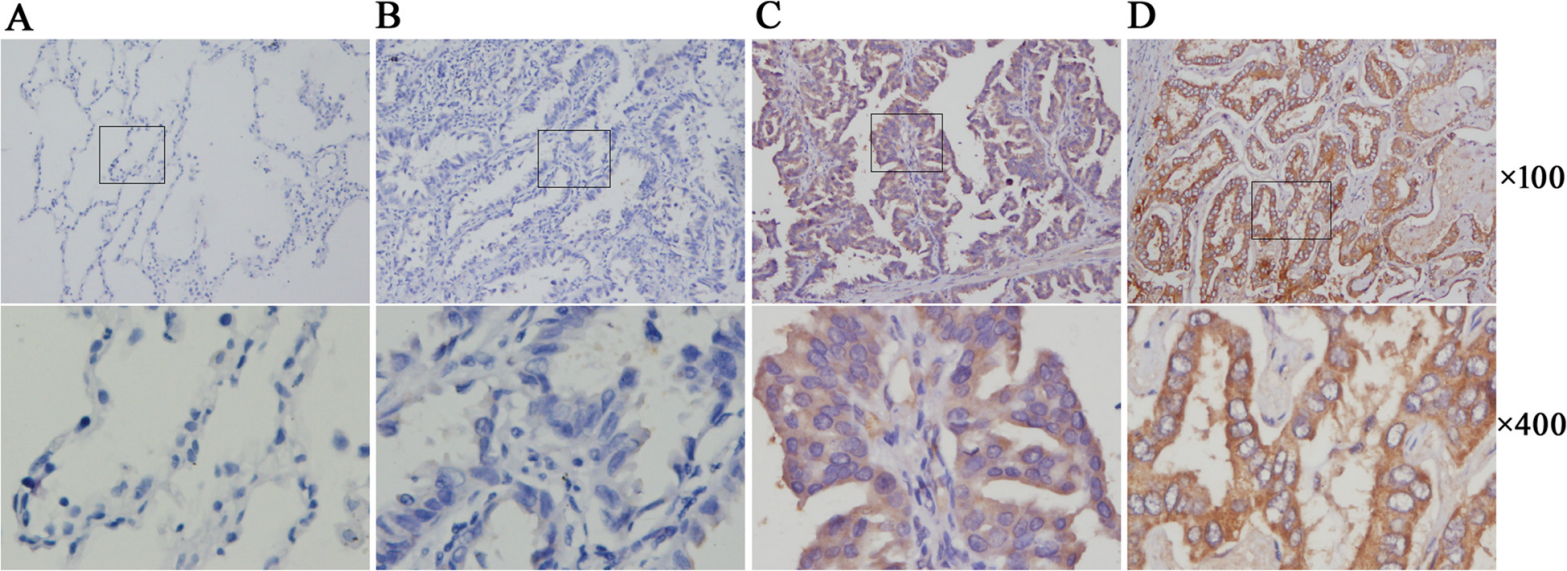
Expression of CCR9 in lung adenocarcinoma and normal lung tissues based on immunohistochemistry. **a** Negative expression in adjacent normal lung tissue; **b** Negative expression in lung adenocarcinoma tissue; **c** Positive expression of CCR9 in lung adenocarcinoma tissue; **d** Strong positive expression of CCR9 in lung adenocarcinoma tissue. CCR9, CC chemokine receptor9

CCR9 expression at the protein level was examined by immunohistochemistry on 144 lung adenocarcinoma tissues and 30 adjacent normal lung tissues (used as normal controls). Figure 1 shows that no CCR9 expression or very weak staining was observed in normal lung epithelia. In contrast, the CCR9 expression was predominantly detected in malignant cells. CCR9 protein was localized in cytoplasm and also on cell membranes, which was clearly stained with deep yellow or brown-yellow. In addition, nuclear CCR9 immunostaining was not observed. High level of CCR9 expression was detected in 105 (72.9 %) of the 144 lung adenocarcinoma samples, whereas it was detected only in 2 (6.7 %) of the 30 adjacent normal lung tissue samples. Table 1 reveals that the positive expression rate of CCR9 was significantly greater in the carcinoma tissue compared with the normal lung parenchymal tissue (p < 0.001).

**Table 1:** Association of CCR9 expression with clinicopathological characteristics from lung adenocarcinoma

Group	Cases	Negative expression n (%)	Positive expression n (%)	*p-value*
Cancer	144	39 (27.1)	105 (72.9)	
Normal lung	30	28 (93.3)	2 (6.7)	< 0.001*
Gender				
Male	95	24 (25.3)	71 (74.7)	
Female	49	15 (30.6)	34 (69.4)	0.494
Ages (years)				
≤ 60	88	22 (25.0)	66 (75.0)	
> 60	56	17 (30.4)	39 (69.6)	0.481
Differentiation				
High	31	12 (38.7)	19 (61.3)	
Moderate	66	17 (25.8)	49 (74.2)	0.225
Low	47	10 (21.3)	37 (78.7)	
Tumor size (cm)				
T1	50	19 (38.0)	31 (62.0)	
> T2	94	20 (21.3)	74 (78.7)	0.032*
Lymph node status				
Yes	68	10 (14.7)	58 (85.3)	
No	76	29 (38.2)	47 (61.8)	0.002*
TNM stage				
Stage I	57	22 (38.6)	35 (61.4)	
Stage II-III	87	17 (19.5)	70 (80.5)	0.012*
Chemotherapy				
Yes	73	23(31.5)	50(68.5)	
No	71	16(22.5)	55(77.5)	0.226

Note: CCR9, CC chemokine receptor 9; TNM, tumor node metastasis classification system; n, number of cells; *p < 0.05

### CCR9 expression in tumor samples and its correlation with clinicopathological characteristics

A total of 144 patients with lung adenocarcinoma were included in our present work. Table 1 summarizes the relationship between CCR9 protein expression and clinicopathological characteristics of the patients with lung adenocarcinoma. According to the classification described in the section of Methods, immunohistochemical analysis clearly showed that the CCR9 expression at the protein level was significantly and positively associated with the tumor size (P = 0.032). CCR9 was also over-expressed in patients with lymph node involvement (N1/2 vs. N0; 85.3 % vs. 61.8 %; p = 0.002). High expression of CCR9 was more prevalent in advanced clinical stage cases compared with early clinical stage cases (80.5 % and 61.4 %, respectively; p = 0.012). In addition, the positive expression rate of CCR9 was the highest in low differentiation subgroup (78.7 %), followed by moderate differentiation subgroup (74.2 %) and high differentiation subgroup (61.3 %). Moreover, a trend was observed between the CCR9 expression and pathologic differentiation, although it was not significant (p = 0.225). Statistical analysis revealed that there was no significant correlation between the CCR9 expression and the age, gender, chemotherapy and differentiation status of patients (p > 0.05).

### OS and prognostic significance of CCR9 expression in lung adenocarcinoma

**Fig. 2:**
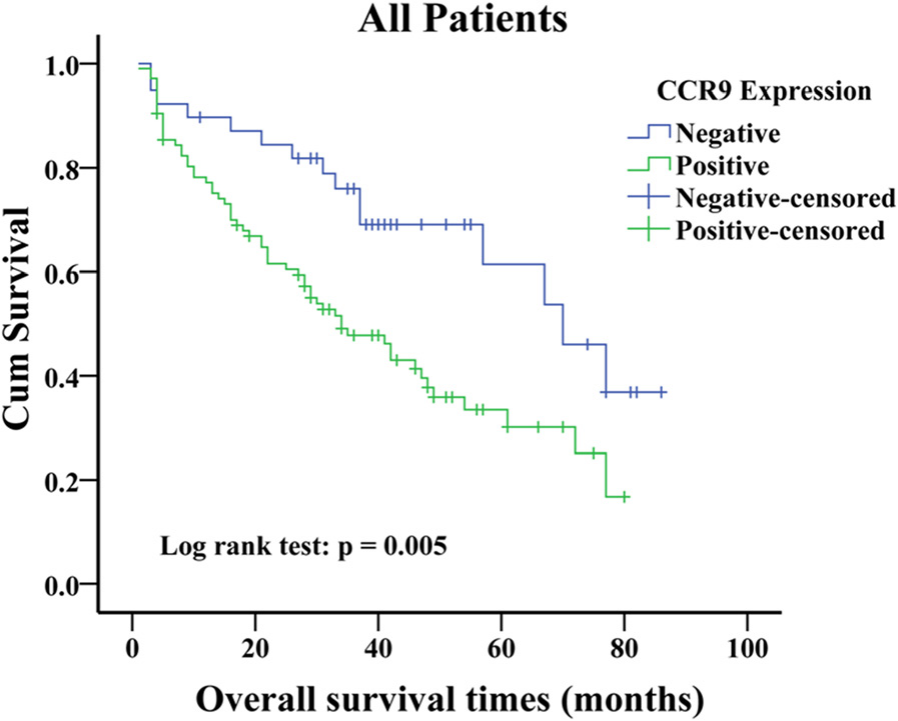
Kaplan-Meier curves of overall survival defined by CCR9 expression (n = 144). Patients with negative CCR9 expression had a greater OS than patients with positive CCR9 expression (Log rank test, P = 0.005). CCR9, CC chemokine receptor9

**Fig. 3:**
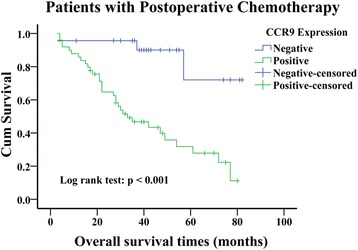
Kaplan-Meier curves of overall survival defined by CCR9 expression in the patients who received postoperative platinum-based chemotherapy (n = 73). Patients who received postoperative chemotherapy with negative CCR9 expression had a greater OS than those with positive CCR9 expression (Log rank test, P < 0.001). CCR9, CC chemokine receptor9

OS analysis using the Kaplan-Meier method showed that patients with negative CCR9 expression had higher OS compared with those with positive CCR9 expression (Fig. 2; log rank value =7.750, P = 0.005). The survival rate of CCR9 negative patients who received postoperative chemotherapy was higher than those received single surgery (Fig. 3; log rank value =14.255, P < 0.001). Moreover, upon the univariate analysis using the cox proportional hazards model, positive lymph node metastasis (HR = 1.71, P = 0.021), advanced TNM stage (HR = 2.184, P = 0.002), postoperative chemotherapy (HR = 0.628, P = 0.044) and positive CCR9 expression (HR = 2.189, P = 0.007) were associated with increased risk of death in the patients with lung adenocarcinoma. In the multivariate analyses, positive CCR9 expression remained predictive for OS after adjustment of lymph node status, TNM stage and chemotherapy as covariates (HR = 1.948, P = 0.028; Table 2). Except for TNM stage, CCR9 expression emerged as an independent prognostic indicator of OS for patients with lung adenocarcinoma.

**Table 2:** Univariate and multivariate analysis of OS in 144 patients with lung adenocarcinoma

Variable	Univariate			Multivariate		
	HR	95 % CI(lower-upper)	*p-value*	HR	95 % CI(lower-upper)	*p-value*
Gender	0.737	0.452-1.204	0.223	/	/	/
Age	0.899	0.564-1.433	0.654	/	/	/
Differentiation	1.121	0.823-1.527	0.468	/	/	/
Tumor size	1.401	0.858-2.288	0.178	/	/	/
Lymph node status	1.710	1.083-2.699	0.021*	0.774	0.387-1.547	0.469
TNM stage	2.184	1.327-3.593	0.002*	2.331	1.117-4.863	0.024*
Chemotherapy	0.628	0.399-0.988	0.044*	0.682	0.431-1.081	0.103
CCR9 expression	2.189	1.237-3.873	0.007*	1.948	1.076-3.526	0.028*

Note: CCR9, CC chemokine receptor 9; TNM, tumor node metastasis classification system; HR, hazard ratio; 95 % CI, means 95 % confidence interval; *p < 0.05

## Discussion

As members of small-molecule superfamily (8–14 kDa), chemokines are classified into four subgroups (CXC, CC, C and CX3C) according to the position of their NH_2_-terminal cysteine residues [3]. It has been known that chemokines and their seven trans-membrane G-protein coupled receptors play roles in regulating the movement of lymphocytes, natural killer cells and dendritic cells under both physiological and pathological conditions. Muller et al. proposed that tumor cells accomplish the process of invasion and metastasis via the chemokine-mediated mechanisms, such as leukocyte trafficking regulation [12]. The aberrant expression of chemokine receptors has been associated with disease severity, increased invasiveness of cancer cells, and even poor prognosis in various types of malignancies [13, 14].

CCR9 is primarily involved in immune system, and it is present on leukocytes and plays critical roles in T lymphocyte development and recruitment of immune cells to the small intestine [15]. Recent data have shown that CCR9 is functionally and significantly expressed in various cancers, such as acute lymphocytic leukemia [16], melanoma [17], prostate cancer [18], pancreatic cancer [19], breast cancer [20], ovarian cancer [21] and colon cancer [22]. In cancer biology, activation of CCR9 by CCL25 promotes cancer cell migration, invasion and expression of matrix metalloproteinases (MMPs), which are key components of cancer invasion and metastasis [23].

In the present study, we analyzed the expression of CCR9 at the protein level in lung adenocarcinoma tissues and adjacent normal lung tissues. Interestingly, CCR9 was expressed at a significantly greater level in tumor tissue compared with normal tissue (p < 0.001). This finding was consistent with our previous experimental result that CCR9 is up-regulated in NSCLC compared with adjacent normal lung tissue at the transcriptional level [10]. Previously, we have also shown that over-expressed CCR9 interacts with CCL25 to promote proliferation and suppress apoptosis of NSCLC cells *in vitro*, and knockdown of CCR9 compromises *in vivo* tumor growth in a nude mice model [11]. These observations demonstrated that like other members of the CC chemokine receptor family [13], CCR9 may function as an oncogene and play an important role in the tumorigenesis and development of lung adenocarcinoma.

Although the prognostic significance of CCR9 expression has not been previously examined in NSCLC, existing evidence suggested that CCR9-CCL25 axis is associated with cancer aggressiveness, and it might be used as a potential prognostic indicator in lung cancer. Clinical and pathological staging of cancer is based on TNM system, which is used to determine the clinical outcome, including therapeutic interventions and prognosis [24]. In principle, larger tumor size (T), positive lymph node metastasis and advanced tumor stage often suggest stronger metastatic capability and poorer prognosis [25]. Gupta et al. have recently reported that CCR9-CCL25 interaction promotes lung cancer progression by facilitating the migration, invasion and metastasis of lung cancer cells, and it selectively modulates the expression of key metastatic factors (MMPs and TIMPs) in lung cancer cells following CCL25 treatment [26]. Furthermore, the number of lung adenocarcinoma cells that migrated and invaded in response to CCL25 is greater compared with lung squamous cell carcinoma cells. The expression and activity of MMP-2 in response to CCL25 were greater in lung adenocarcinoma cells compared with squamous cell carcinoma cells [26]. Interestingly, our current founding showed that higher expression of CCR9 could be linked with tumor size in lung adenocarcinoma; the positive expression rate of CCR9 (58/68, 85.3 %) in tumor tissue with lymph node metastasis was greater than that without lymph node metastasis (47/76, 61.8 %). Moreover, higher expression of CCR9 in lung adenocarcinoma was significantly correlated with advanced TNM stage (p = 0.012). In addition, we observed a clear trend between CCR9 expression and pathologic differentiation, although this trend was not significant (p = 0.225). In the previous study, we have found that CCR9-CCL25 axis plays a significant role in survival of NSCLC cells by inhibiting cancer cell apoptosis both *in vitro* and *in vivio* through the activation of PI3K/Akt signaling pathway, and cell survival is essential for cancer cells to achieve invasion and metastasis [11]. Therefore, over-expression of CCR9 in lung adenocarcinoma suggested its crucial role in disseminating primary tumor and promoting tumor cell survival during metastasis. Li et al. reported that high CCR9 expression is detected in tumor tissue and tumor draining lymph nodes in patients with pancreatic cancer, which is positively associated with cancer progression and metastasis [27]. These results indicated that the CCR9 expression might be predictive for lymph node metastasis in patients with lung adenocarcinoma, therefore more aggressive treatment should be considered in the patients at high risk of lymph node metastasis.

Zhang et al. reported that ectopic expression of CCR9 not only enhances cell proliferation and tumorigenicity in hepatocellular carcinoma cells, but also acts as a novel prognostic marker and therapeutic target for hepatocellular carcinoma [9]. Similarly, Li et al. demonstrated that positive expression of CCR9 is significantly correlated with poor prognosis in patients with pancreatic cancer [27]. Nevertheless, it remains elusive whether these findings of CCR9 can be extended to lung adenocarcinoma patients. In this study, we, for the first time, reported the prognostic value of CCR9 expression for postoperative patients with lung adenocarcinoma. According to Kaplan-Meier survival analysis, expression of CCR9 was negatively associated with OS in lung adenocarcinoma, as the patients with positive CCR9 expression exhibited a poorer OS. Johnson et al. demonstrated that ovarian cancer cells and tissue express CCR9, and the interaction between CCR9 and CCL25 increases anti-apoptotic signaling cascades in ovarian cells, which rescues cells from cisplatin-induced death [28]. The similar result has been observed in breast cancer *in vitro* that CCR9-CCL25 axis inhibits cisplatin-induced apoptosis in a PI3K-dependent and FAK-independent fashion [29]. Although no previous study has investigated the relationship between CCR9 and chemotherapy in lung cancer, interestingly, among the lung adenocarcinoma patients who received postoperative platinum-based chemotherapy, the patients with negative CCR9 expression had a significant better prognosis. It is necessary to further clarify the underlying molecular mechanism of CCL25-CCR9 axis on lung adenocarcinoma. Furthermore, after adjustment of lymph node status, TNM stage and chemotherapy, multivariate analysis revealed that CCR9 expression could be used as an independent factor for predicting post-operation survival in patients with lung adenocarcinoma.

There were limitations of this study. Firstly, the sample size was still small, and the uneven distribution of each subgroup, including the TNM staging of these patients, might have biased this result. Secondly, many factors may affect the prognosis of the patients with lung adenocarcinoma after operation, and there was possible residual confounding in this study.

Taken together, we, for the first time, investigated the prognostic impact of CCR9 on lung adenocarcinoma. The expression of CCR9 was significantly increased in lung adenocarcinoma tissue. Such an increased CCR9 expression was associated with larger tumor size, positive lymph node metastasis, advanced clinical stage and poor prognosis among patients with lung adenocarcinoma, suggesting that CCR9 might be a novel prognostic biomarker for lung adenocarcinoma.

## Conclusion

These results suggest for the first time that CCR9 expression may be beneficial in predicting lymph node metastasis and survival of patients with lung adenocarcinoma. A future study will investigate whether CCR9 can act as a new therapeutic target in lung adenocarcinoma.
